# A prospective cohort study of outcomes for isoniazid prevention therapy: a nested study from a national QI collaborative in Uganda

**DOI:** 10.1186/s12981-020-00285-0

**Published:** 2020-05-27

**Authors:** Simon Sensalire, Esther Karungi Karamagi Nkolo, Juliana Nabwire, Anna Lawino, Dithan Kiragga, Martin Muhire, Herbert Kadama, Cordelia Katureebe, Proscovia Namuwenge, Joshua Musinguzi, Jacqueline Calnan, Dejene Seyoum

**Affiliations:** 1 University Research Co., LLC, USAID RHITES N-Acholi Activity, Kampala, Uganda; 2 Ministry of Health, AIDS Control Program, Kampala, Uganda; 3 United States Agency for International Development (USAID), Kampala, Uganda

**Keywords:** IPT, Completion, Collaborative, Quality improvement

## Abstract

**Background:**

Tuberculosis (TB) and human immunodeficiency virus (HIV) co-infection constitute a deadly infectious disease synergy disease and major public health problem throughout the world. The risk of developing active TB in people living with HIV (PLHIV) is 21 times higher than the rest of the world population. The overlap of latent TB infection and HIV infection has resulted in marked increases in TB incidence in countries with dual epidemics. Although antiretroviral therapy (ART) is the single most significant way to reduce incident TB in PLHIV, besides early ART initiation, isoniazid preventive therapy (IPT) is the key intervention to prevent TB among PLHIV. This prospective cohort and longitudinal study aimed to document; retention, adherence, development of active TB disease, possible adverse drug reactions and completion among patients initiated on IPT in Jan 2019.

**Methods:**

This was both a prospective cohort and longitudinal study nested within a national quality improvement collaborative in which multiple quality improvement teams tested changes in care delivery to improve the delivery of IPT. The prospective cohort were HIV patients without TB disease initiated on a dosage of Isoniazid 300 mg/day for adults and 150 mg/day for children for a period of 6 months. Association statistics were used to describe patient characteristics and outcomes. Variables with p-value < 0.05 were used to determine linear by linear associations between patient characteristics assumed to influence both primary and secondary outcomes. Variables with a p-value < 0.05 were included in the logistical regression model. The final model included those factors that retained statistical significance. The odds ratios (OR) and adjusted OR (AOR) along with its 95% confidence interval were used to determine the power of relationship in determining the outcomes of interest. The model was tested for fitness using goodness-of-fit Hosmer–Lemeshow tests.

**Results:**

The completion of IPT was at 89%. A significant proportion of patients adhered to treatment (89%) and kept their appointment schedules-retention (89%). All patients (100%) received IPT at each appointment visit. Only 4% of patients experienced side effects of isoniazid (INH) but none of them developed active TB at the end of the 6 month INH dose. Multivariate logistic regression analysis of covariates of IPT completion revealed a strong and statistical association between IPT completion and age, gender, retention and side effects of INH. Our multivariate model found that children below 15 years were less likely to complete INH than patients ≥ 15 years (*AOR *= 0.416, *p *= 0.230, *df *= 1). Female patients were 2 times more likely to complete INH dose than male patients (AOR = 1.598, *p *= 0.018). Patients who kept all their appointment schedules were 10 times more likely to complete IPT than those who missed one or more schedules (*AOR *= 10.726, *p *= 0.000, *df *= 1). We also found that patients who did not report any side effects associated with INH were 2 times more likely to complete INH (*AOR *= 1.958, *p *= 0.016, *df *= 1) than patients who reported one or more side effects.

**Conclusion:**

Treatment completion is the end-point of the IPT initiation strategy in Uganda. With a completion rate of 89%, our results seem re-assuring and suggest that improvement collaborative is an effective approach to achieving results through combined efforts. The high rates of completion are encouraging indicators of progress in the implementation of collaborative activities in the study setting. However, such collaboratives would require periodic evaluation to prevent possible relapses in progress attained.

## Background

Tuberculosis (TB) and HIV make up a deadly synergy of infectious disease, and the combined effect is apparent in resource-limited countries like Uganda. TB remains a main public health problem throughout the world, even with the recent advancement of extensive global TB control efforts [1]. According to the World Health Organization (WHO) 2017 report, there are 10.4 million incident cases of TB, out of which an estimated 1.04 million (10%) occurred in HIV positive patients. The risk of developing active TB in people living with HIV (PLHIV) is 21 times higher than the rest of the world population [2]. The overlap of latent tuberculosis infection (LTBI) and HIV infection has resulted in marked increases in TB incidence in countries with dual epidemics and TB has become one of the most common opportunistic infections and the leading cause of death in HIV-infected people in Africa, Asia and Latin America [3].

Although antiretroviral therapy (ART) is the single most significant way to reduce incident TB in PLHIV, HIV positive patients receiving ART remain very susceptible to TB. Therefore, besides early ART initiation, isoniazid preventive therapy (IPT) is the key intervention to prevent TB among PLHIV [3]. Regarding the concomitant use of IPT with ART, recent evidence revealed that the combined use of IPT and ART have a synergetic effect in averting incident TB among HIV positive patients [3].

The World Health Organization recommends IPT/TB Preventive Treatment (TPT) to treat tuberculous infection in high-risk populations, such as persons living with human immunodeficiency virus (PLHIV) and children aged 5 years who are close contacts of patients with infectious TB [4]. In 2014, Uganda adopted the WHO guidelines for Intensified TB Case finding (ICF) and IPT/TPT as part of the comprehensive HIV/AIDS care strategy [5].

While there is adequate evidence that the use of IPT results in a lower risk of progression to TB disease and TB related mortality for people with HIV, it is important to document completion. In contexts where patients on ART are in different service delivery models, completion of IPT is of concern and must be prioritized. In Uganda, a national quality improvement (QI) collaborative was used to address multiple health care delivery, patient and provider level barriers to IPT completion using the continuous quality improvement (CQI) approach. In a collaborative, multiple teams work toward the same aims and share their results so all teams can learn how to most efficiently improve care. Researchers must recognize the need to evaluate not only endpoint outcomes, but also assess the extent to which implementation is effective in a specific context to optimize intervention benefits, prolong the sustainability of the intervention in that context, and promote the dissemination of findings into other contexts [6].

A national QI collaborative to improve IPT completion was initiated in January 2019 as part of a wider continuous quality improvement effort at scale in Uganda. The collaborative covered all the 14 health regions of Uganda, 126 out of 127 districts and 739 ART accredited sites. It entailed situational analysis of IPT completion in high volume facilities and coaching support to sites in all regions. The situation analysis helped to identify gaps and to come up with changes/innovations for IPT completion. Regional coaches supported district-based coaches to support site teams to understand underlying factors for low IPT completion, identify interventions for improving IPT completion and develop improvement action plans. Depending on context and informed by root cause analysis, different changes for improvement were tested to achieve adherence and completion for all patients on TPT in each facility. The site teams reviewed progress in addressing barriers to the completion of INH. We present outcomes from a prospective and longitudinal cohort study nested within the national QI collaborative.

### The intervention

The intervention to improve IPT completion was part of a wider continuous quality improvement effort at scale in Uganda following the roll out of the consolidated guidelines for prevention and treatment of HIV in Uganda. The intervention was implemented as a national QI collaborative since January 2019 and covered all regions of Uganda. The intervention was led by the AIDS Control Program, Ministry of Health (MoH) while USAID RHITES N-Acholi provided QI technical support. The PEPFAR implementing partners (IPs) rolled out the collaborative activities for IPT through the regional and district coaches. Regional and district coaches were identified among public health workers by IPs and District Health Teams with a basic selection criterion being public health worker with knowledge or previous experience in QI for HIV. The coaches were trained in QI and their roles followed by monthly coaching visits to health facilities to support improvement activities for IPT among other interventions such as viral load suppression and retention. The coaches supported sites quality improvement teams (QITs) to identify and address gaps for IPT and review progress in addressing barriers to the completion of INH. document improvements in initiation and completion. Depending on context and root cause analysis, different changes for improvement were tested to address gaps for IPT. Quarterly review meetings were held for IPs and regional coaches by MoH to share experiences and spread learning. District learning sessions were organized by IPs for health facilities. We present outcomes from a prospective longitudinal cohort of patients initiated on IPT in January 2019. The purpose of this study was to document retention, adherence, development of active TB disease, possible side effects and completion. For study purposes, the primary outcomes were IPT completion defined as having received and consumed a 6-month course of INH and the development of active TB disease.

## Methods

### Study subjects

The prospective cohort were patients on ART without active TB disease who were initiated on IPT in January 2019. Isoniazid was administered at a dose of 300 mg/day for adults and 150 mg/day for children for a period of six months. Pyridoxine (vitamin B6) was given to patients to prevent possible side effects of INH.

To rule out active TB disease among patients, screening for active TB was conducted as part of routine clinical care using clinical assessment with the aid of the intensive TB case finding guide that was administered to all study participants on INH at each clinic visit. Patients who were identified with presumptive TB were further investigated using microscopy, GeneXpert (Xpert MTB/RIF assay) and X-ray depending on the symptoms of presentation. Microscopy or GeneXpert was used for PLHIV presumptive TB patients with no danger signs and with CD4 > 200. X-ray was used for clients from whom samples could not be obtained and from clients with negative microscopy or GeneXpert results.

### Study design

This was both a prospective cohort and longitudinal study nested within the national QI collaborative in which multiple QITs tested changes in care delivery to improve the delivery of IPT. The study was conducted in ART clinics in 14 high volume sites. High volume sites were operationally defined as sites handling approximately 2000 patients in the outpatient department. The study sites were public facilities and represented all levels of health facility. Study sites were supported to commit full doses of INH and Pyridoxine (vitamin B6) for study participants up to completion to prevent stock out. Additional support was given to study sites on the management of uncommitted doses of INH and Pyridoxine (vitamin B6) for new patients as an added benefit of the study to the study sites.

### Data collection

The patients initiated on INH were monitored consecutively for 6 months for retention, adherence, development of active TB disease, possible adverse drug reactions and completion. Data for all patients initiated on IPT in the January cohort was captured and continuously updated monthly by health workers up to 7 months during when the cohort would have completed IPT. Both primary and secondary outcomes were captured during and at the end of the study for each of the patients in the cohort. The primary outcome was IPT completion defined as completing a 6-month course of INH within 6 months. Secondary outcome measures are adherence and retention in care.

### Data analysis

Frequency and proportions were obtained to describe patient characteristics such as age, gender and outcome variables: retention, adherence, side effects, and completion. Association statistics were used to determine linear by linear associations between patient characteristics which, from a theoretical point of view, were assumed to influence both primary and secondary outcomes. Variables with p-value < 0.05 were included in the logistical regression model using a backward stepwise method. The final model included those factors that retained statistical significance. The odds ratios (*OR*) and adjusted odds ratio (*AOR*) along with its 95% confidence interval (*CI*) were used to determine the power of relationship in determining the outcomes of interest. Results are presented as *AOR* with 95% confidence intervals, and level of significance at 5% (*p *< 0.05). The model was tested for fitness using the goodness-of-fit Hosmer–Lemeshow tests.

### Ethical considerations

This study was reviewed and approved in accordance with the guidelines for conducting research. It was reviewed and approved by a local institutional review board and Ministry of Health.

## Results

### Patients characteristics

Patients were recruited in January 2019 upon initiation on INH and followed up to the end of 6 months. Patients initiated after January 2019 were excluded from the study. One thousand and twenty-six patients (1026) patients were ultimately considered for the study in fourteen (14) high volume public health sites from different regions. Sixteen (16) patients were excluded in the analysis at the end of the 6 months period for lack of documented outcomes. We documented both secondary and primary outcomes at the end of 6 months of initiation on INH for 1010 patients.

Most patients were adults ≥ 15 years (94%) and 6% were children below 15 years. The median age for the cohort was 39. Females predominated patients at 61% compared to 39% of males. The majority of patients were on 300 mg dose per day on account of their age and weight. Child dose factored age and weight. All patients had a known HIV positive status and were on antiretroviral therapy (100%). The proportions of the patient characteristics and their corresponding numbers (n) are shown in Table 1.

**Table 1:** Patient characteristics

Patient characteristics	Numbers (n)	Percentages (%)
Age (years)		
< 15	56	6.0
≥ 15	954	94.0
Total	1010	100
Gender		
Female	621	61.4
Male	389	38.5
Total	1010	100
INH dose (mg/day)		
150	56	6.0
300	954	94.0
Total	1010	100
HIV status		
Positive	1010	100
Negative	0	0
Total	1010	100
ART status		
Already on ART	1010	0
Not on ART	0	0
Total	1010	100

### Secondary outcomes of IPT

This study sought information on secondary outcomes including; appointment keeping-retention, adherence to INH and side effects of INH. The results are contained in Table 2; results for each of the secondary outcomes is described as follows:

**Table 2:** Secondary outcomes for Isoniazid Prevention Therapy

Patient characteristics	Numbers (n)	Percentages (%)
Kept all appointments-retention		
Yes	903	89.4
No	107	10.6
Total	1010	100
Appointment keeping over the study period		
First appointment	1007	99.7
Second appointment	1001	99.1
Third appointment	979	97.0
Fourth appointment	966	95.6
Fifth appointment	950	94.0
Sixth appointment	923	91.3
Received TPT at each appointment		
Yes	903	89.4
No	107	10.6
Total	1010	100
Had good adherence score of ≥ 95% at each appointment		
Yes	899	89.0
No	111	11.0
Total	1010	100
Experienced side effects from INH		
Yes	41	4.0
No	969	96.0
Total	1010	100

### Six-month trends in appointment keeping and TPT

In this study, appointment keeping was estimated as the proportion of individuals who kept each appointment. In this study, appointment keeping was used synonymously with retention. Appointment keeping at all scheduled visits was 89%. Individual appointments per month averaged at 96% and was generally higher within the first 3 scheduled appointments. TPT was given to all patients at each scheduled appointment visit (100%).

### Adherence to INH

In this study, adherence was a secondary outcome. Results in Table 2 shows that eighty-nine percent (89%) of patients had a good adherence score of ≥95% on all appointment schedules. Adherence scores were estimated from pill counts of INH drugs since the last appointment visit with the patient.

### Side effects of INH

The study determined whether patients experienced any side effects of INH such as numbness, tingling and burning sensation, blurred or loss of vision, convulsions, mood changes, unusual bleeding, skin rash, sore throat, joint pain, fever, dizziness, loss of appetite, nausea and vomiting, and dark urine. Results in Table 2 shows that only 4% of patients reported a side effect associated with INH.

### Primary outcomes of IPT

The primary outcome for this study was the completion of a six-month dose of INH and the development of active TB. None of the patients developed active TB disease over the six-month period and excludes the 2% (n = 17) patients who were lost to follow up.. Completion of a six-month adult (300 mg) and child dose (150 mg) of INH was 89%, confirmed on the 7th appointment when all medicine was taken as prescribed. Nine percent (9%, n = 92) of patients stopped treatment and were excluded from analysis. Of these (55%, n = 51) stopped INH treatment because of ARV treatment interruption. The rest of the patients stopped treatment due to side effects (45%, n = 41). There were no deaths reported during the 6 months of INH. The results showing the primary outcomes for IPT are contained in Table 3.

**Table 3:** Primary outcomes for isoniazid prevention therapy

Patient characteristics	Numbers (n)	Percentages (%)
Developed active TB disease over six months period^a^		
Yes	0	0
No	993	100
Primary outcome^b^		
Completed	901	89.2
Never completed/unknown outcome	109	10.8
Total	1010	100
Other outcomes^c^		
Lost to follow up	17	1.7
Stopped treatment	92	9.1
Unknown	16	1.6
Died	0	0
Reason for stopping^d^		
Side effects	41	44.6
Treatment interruption	51	55.4
Total	92	100

^a^Only patients with known outcome and includes those who completed and those who stopped treatment

^b^Excludes patients with unknown outcome

^c^Excludes patients who completed treatment and is expressed out of the total cohort of 1026

^d^Includes only patients who stopped treatment

### Covariates of completion of IPT

Multivariate logistic regression analysis of covariates of IPT completion revealed a strong and statistical association between IPT completion and age, gender, keeping appointments and side effects of INH. We found that completion of INH was statistically significantly related to gender in our multivariate model. Results showed that female patients were 2 times more likely to complete an INH dose than male patients (*AOR *= 1.598, *p *= 0.018). Children below 15 years were less likely to complete INH than patients ≥ 15 years (AOR = 0.416, *p *= 0.230, df = 1). Patients who kept all their scheduled appointment were 10 times more likely to complete IPT than those who missed one or more schedules (*AOR *= 10.72, *p *= 0.000, df = 1). In our analysis, patients who did not report any side effects associated with INH were 2 times more likely to complete INH than patients who reported one or more side effects associated with INH (*AOR *= 1.958, *p *= 0.016, df = 1). Adherence which was operationally defined as taking drugs as prescribed by the health worker was a predictor of completion. Patients who had an adherence score of ≥ 95% were 2 times more likely to complete treatment within 6 months (AOR = 1.526, p = 0.013, df = 1) than those with fair adherence (≥ 85%). The results showing covariates of completion are contained in Table 4.

**Table 4:** Covariates of completion of isoniazid prevention therapy

Variable	*Coefficient*	*p*-*value*	*df*	*AOR*	*95% CI*
Age (years)					
< 15	− 0.877	0.230	1	0.416	(0.099–1.745)
≥ 15	0.000	–		1.000	
Gender					
Females	0.469	0.018	1	1.598	(0.896–2.470)
Male	0.000	–		1.000	
Kept all appointments					
Yes	2.373	0.000	1	10.726	(0.112–0.791)
No	0.000	–		1.000	
Side effects					
No	0.672	0.016	1	1.958	(0.17–1.393)
Yes	0.000	–		1.000	
Adherence score ≥ 95%					
Yes	0.423	0.013	1	1.526	(0.647–1.644)
No	0.000	–		1.000	

## Discussion

The implications of our assessment are multi-fold as it provides a synthesis of primary and secondary outcomes for IPT in the context of a limited resource setting. The study was conducted in a setting where collaborative initiatives used quality improvement approaches to reach the UNAIDS 90-90-90 targets and sustain epidemic control relying on the WHO guidelines for the public health approach. Several studies have assessed completion of INH. All these studies have provided information critical for determining covariates of treatment outcomes but not displayed a nationally representative assessment of TPT outcomes from a perspective of a national collaborative. This study was nested on the national collaborative aimed at improving IPT completion as one of the priority areas.

We reported a completion rate of 89% for a cohort of patients initiated on a six month INH dose. The results are in tandem with what other studies have documented in similar jurisdictions in Addis Ababa in Ethiopia which showed an adherence level of 89.5% [7] The findings are expected in our case given improvement interventions for IPT completion in the study sites under the national QI collaborative. However, we were unable to control for other covariates to IPT completion given that the study was nested on the national QI collaborative.

Other jurisdictions have documented a lower completion rate. A study conducted by TRAM et al. [8] found that 73% of patients completed IPT. However, results in this study indicate 89% completion rates at a more national representative level among people who are retained on IPT and on ART. Indeed, because of the longitudinal and prospective design, it adds another degree of precision of results which are potentially associated with improvement activities for the national collaborative that focused IPT as one of the priority areas in the study sites.

Our results showed that among both children and adults, the proportion of patients who completed 6 months of IPT was (> 89%). Similar findings were documented in the study in Congo on the high completion of Isoniazid Preventive Therapy among HIV-infected children and adults. The study found more than 85% completion on INH for both children and adults [9]. Our findings are further corroborated by other studies which have documented completion of other medication. Our finding that completion was 89% is almost similar to that observed in a TB vaccine trial in Tanzania [10] and in the public primary HIV clinics in Brazil (85%) [11].

Adhering to preventive medical regimens can be difficult, especially when patients feel well, and regimens require months to complete. Reported adherence rates to TPT are highly variable in adults, pregnant women, and children living with HIV, and range from under 37% to greater than 95% [12]. Similar observations were documented in our study with adherence to 89%. The consequences of this adherence level were essential that patients were protected against the possibility of acquiring active TB disease during or at the end of the treatment. In our study outcomes, none of the patients developed active TB at the end of the six months of INH.

In a related analysis, poor adherence limits virologic control and a loss of immunological and clinical improvement [13]. Our findings corroborate this study where we also observed that patient’s development of active TB disease was prevented because adherence level was 89%. Adherence counseling before IPT along with adherence monitoring for ART was one way to determine readiness to start and complete treatment. Although patients with slightly less than 95% adherence completed, their treatment duration was elongated proportionate to the missed doses before completion outcome was entered for the patient. In this study, adherence was operationally defined to mean taking drugs every day as prescribed by the health worker.

We investigated the possible side effects of INH in this cohort study and found no close association with the completion of the INH dose. Clinicians and program personnel frequently express concern about the potential toxicity of TPT medications, especially for patients who are also receiving ART. Neuropathy is associated with INH but is also seen with certain antiretrovirals (ARVs) and HIV infection itself, making it difficult sometimes to determine the causative agent. It is more frequent in PLHIV who are undernourished and can be prevented with concurrent prescription of pyridoxine (vitamin B6) [14]. Blurred or loss of vision, convulsions, mood changes, unusual bleeding, skin rash, sore throat, joint pain, fever, dizziness, loss of appetite, nausea and vomiting, dark urine were among other side effects that we are monitoring in the course of the study. However, there was limited data given the rarity of clinically relevant adverse events and effects. The study did not document baseline side effects prior to TPT initiation to conclude potential side effects associated with TPT.

This study was nested to a QI intervention which aimed at improving provider skills, initiating patients without active TB on INH and preventing stock outs of INH at health facilities. Previous studies have closely linked programmatic issues to adherence, completion, and management of side effects among patients on TPT such as health provider capacity to diagnose and manage patients and stock outs. A study conducted in western Uganda showed shows that it is not difficult to link the insufficiencies in the health care system with the poor outcome of TB patients. The study found that the lack of health care worker training resulted in extensive delays in diagnosis and management of TB/HIV co-infected patients [15]. This suggest the need for depth training for health care workers, especially at primary health centers to better identify TB and manage side effects.

The under-performance of supply chains presents a significant hindrance to disease control in developing countries. Stock-outs of essential medicines lead to treatment interruption which can force changes in patient drug regimens, drive drug resistance and increase mortality. In the case of TB, stock-outs of anti-tuberculosis drugs lead to treatment interruption which can force changes in patient drug regimens, drive drug resistance and increase mortality [16].

This study had some limitations. There was no control group, to ascertain what happens where no initiatives for improving completion are in place. Thus, the analysis may have bias arising from studying patients from sites with ongoing improvement initiatives for IPT but was cured by creating cohorts with a baseline month when all factors were considered the same. Secondly, adherence was measured by pill counts which may not reflect actual patient adherence practices. However, the study established patient’s clinical attendance which could be used as a proxy indicator of the patient’s intention to comply with treatment. Our cohort was part of the HIV-infected children and adults receiving IPT as part of their routine HIV care and treatment and therefore could not determine whether being on ART was a predictor of completion. The findings are, therefore, generalizable in Uganda and other resource-limited settings.

## Conclusion

Treatment completion is the end-point of the IPT initiation strategy in Uganda. With an 89% completion rate, our results are reassuring but call for more concerted efforts to achieve over 95% target. The 89% completion rates in the national QI sites suggest a collaborative as effective in achieving high uptake of preventive interventions at scale in the study setting. However, the ongoing delivery of IPT would require periodic evaluation to prevent possible relapses in progress attained. The recognition that IPT provision for PLHIV is bound to challenges resulting from the covariates of completion implies a need for more serious commitments to address known and anticipated barriers for the evidence-based method of prevention to benefit-eligible patients. Studies such as this one, are necessary for routine monitoring of successes to prevent possible relapses in both secondary and primary outcomes so far documented.

## Data Availability

The datasets generated during the study are not publicly available due to some personal information of patients but are available from the corresponding author on reasonable request.

## Abbreviations

ART: Antiretroviral therapy
ARVs: Antiretroviral drugs
AIDS: Acquired immune deficiency syndrome
AOR: Adjusted odds ratio
CI: Confidence interval
CQI: Continuous quality improvement
IPT: Isoniazid preventive therapy
IP: Implementing partner
INH: Isoniazid/isonicotinylhydrazide
LTBI: Latent tuberculosis infection
MoH: Ministry of Health
HIV: Human immunodeficiency virus
PEPFAR: President’s Emergency Plan for AIDS Relief
PLHIV: People living with HIV
PMTCT: Prevention of mother-to-child transmission
QI: Quality improvement
QITs: Quality improvement Teams
RHITES: Regional Health Integration to Enhance Services
TB: Tuberculosis
TPT: Tuberculosis preventive therapy
UNAIDS: Joint United Nations Programme on HIV/AIDS
USAID: United States Agency for International Development
WHO: World Health Organization
UNCST: Uganda National Council of Science and Technology

## References

[1] Sulis G, Roggi A, Matteelli A, Raviglione MC (2014). Tuberculosis: epidemiology and control. Mediterr J Hematol Infect Dis..

[2] World Health Organization. Global tuberculosis control. WHO report 2017. https://www.who.int/tb/publications/global_report/gtbr2017. Accessed 2 Aug 2017.

[3] Corbett EL, Watt CJ, Walker N, Maher D, Williams BG, Raviglione MC, Dye C (2013). The growing burden of tuberculosis: global trends and interactions with the HIV epidemic. Arch Intern Med.

[4] World Health Organization. Guidelines on the management of latent tuberculosis infection. Geneva: WHO; 2015 (http://www.who.int/tb/publications/ltbidocument_page/en/. Accessed 30 Aug 2016. 25973515

[5] National Policy and Guidelines for TB/HIV Collaborative Activities in Uganda. Ministry of Health, Uganda. https://www.who.int/hiv/pub/guidelines/uganda.pdf. Accessed 14 Oct 2016.

[6] Stetler CB, Legro MW, Wallace CM, Bowman C, Guihan M, Hage-dorn H, Kimmel B, Sharp ND, Smith JL (2006). The role of formative evaluation in implementation research and the QUERI experience. J Gen Intern Med.

[7] Berhe M, Demissie M, Tesfaye G (2014). Isoniazid preventive therapy adherence and associated factors among HIV positive patients in Addis Ababa. Ethiopia Adv Epidemiol..

[8] Tram KH, Mwangwa F, Atukunda M, Owaraganise A, Ayieko J, Plenty A, Kwariisima D, Clark TD, Petersen ML, Charlebois ED, Kamya MR, Chamie G, Havlir DV, Marquez C (2017). search collaboration isoniazid preventive therapy completion in the era of differentiated HIV care. PMJ Acquir Immune Defic Syndr..

[9] Yotebieng M, Edmonds A, Lelo A, Wenzi LK, Ndjibu PT, Lusiama J, Kabuayi JP, Behets F (2015). High completion of Isoniazid Preventive Therapy among HIV-infected children and adults in Kinshasa, Democratic Republic of Congo. AIDS..

[10] Munseri PJ, Talbot EA, Mtei L, Fordham von Reyn C (2008). Completion of isoniazid preventive therapy among HIV-infected patients in Tanzania. Int J Tuberc Lung Dis..

[11] Durovni B, Cavalcante SC, Saraceni V, Vellozo V, Israel G, King BS, Cohn S, Efron A, Pacheco AG, Moulton LH, Chaisson RE, Golub JE (2010). The implementation of isoniazid preventive therapy in HIV clinics: the experience from the TB/HIV in Rio (THRio) study. AIDS..

[12] Nanoo A, Izu A, Ismail NA, Ihekweazu C, Abubakar I, Mametja D (2015). Nationwide and regional incidence of microbiologically confirmed pulmonary tuberculosis in South Africa, 2004–12: a time series analysis. Lancet Infect Dis..

[13] El-Khatib Z (2010). Viremia and drug resistance among HIV-1 patients on antiretroviral treatment: a cross-sectional study in Soweto, South Africa. AIDS..

[14] Van der Watt JJ, Harrison TB, Benatar M, Heckmann JM (2011). Polyneuropathy, anti-tuberculosis treatment and the role of pyridoxine in the HIV/AIDS era: a systematic review. Int J Tuberc Lung Dis..

[15] Wynne Ashley, Richter Solina, Banura Lilian, Kipp Walter (2014). Challenges in tuberculosis care in Western Uganda: health care worker and patient perspectives International. J Afr Nurs Sci.

[16] Bam L, McLaren ZM, Coetzee E, Von Leipzig KH (2017). Reducing stock-outs of essential tuberculosis medicines: a system dynamics modelling approach to supply chain management. Health Policy Plann.

